# Patient-important outcomes in randomized controlled trials in critically ill patients: a systematic review

**DOI:** 10.1186/s13613-017-0243-z

**Published:** 2017-03-07

**Authors:** Stéphane Gaudry, Jonathan Messika, Jean-Damien Ricard, Sylvie Guillo, Blandine Pasquet, Emeline Dubief, Tanissia Boukertouta, Didier Dreyfuss, Florence Tubach

**Affiliations:** 1 Service de Réanimation Médico-Chirurgicale, Hôpital Louis Mourier, AP-HP, 178 rue des Renouillers, 92700 Colombes, France; 2 ECEVE, UMRS 1123, Sorbonne Paris Cité, Univ Paris Diderot, Paris, France; 3 ECEVE, U1123, CIC 1421, INSERM, Paris, France; 4 IAME, UMR 1137, INSERM, 75018 Paris, France; 5 IAME, UMR 1137, Sorbonne Paris Cité, Univ Paris Diderot, 75018 Paris, France; 6 Département de Biostatistiques, Santé Publique et Informatique Médicale, CIC 1421, Hôpital Pitié Salpétrière, AP-HP, Paris, France; 7 Unité de Recherche Clinique Paris Nord, Hôpital Bichat, AP-HP, Paris, France; 8 Université Pierre et Marie Curie, Sorbonne Universités, Paris, France

**Keywords:** Patient-important outcome, Critical care, Quality of life

## Abstract

**Background:**

Intensivists’ clinical decision making pursues two main goals for patients: to decrease mortality and to improve quality of life and functional status in survivors. Patient-important outcomes are gaining wide acceptance in most fields of clinical research. We sought to systematically review how well patient-important outcomes are reported in published randomized controlled trials (RCTs) in critically ill patients.

**Methods:**

Literature search was conducted to identify eligible trials indexed from January to December 2013. Articles were eligible if they reported an RCT involving critically ill adult patients. We excluded phase II, pilot and physiological crossover studies. We assessed study characteristics. All primary and secondary outcomes were collected, described and classified using six categories of outcomes including patient-important outcomes (involving mortality at any time on the one hand and quality of life, functional/cognitive/neurological outcomes assessed after ICU discharge on the other).

**Results:**

Of the 716 articles retrieved in 2013, 112 RCTs met the inclusion criteria. Most common topics were mechanical ventilation (27%), sepsis (19%) and nutrition (17%). Among the 112 primary outcomes, 27 (24%) were patient-important outcomes (mainly mortality, 21/27) but only six (5%) were patient-important outcomes besides mortality assessed after ICU discharge (functional disability = 4; quality of life = 2). Among the 598 secondary outcomes, 133 (22%) were patient-important outcomes (mainly mortality, 92/133) but only 41 (7%) were patient-important outcomes besides mortality assessed after ICU discharge (quality of life = 20, functional disability = 14; neurological/cognitive performance = 5; handicap = 1; post-traumatic stress = 1). Seventy-three RCTs (65%) reported at least one patient-important outcome but only 11 (10%) reported at least one patient-important outcome besides mortality assessed after ICU discharge.

**Conclusion:**

Patient-important outcomes are rarely primary outcomes in RCTs in critically ill patients published in 2013. Among them, mortality accounted for the majority. We promote the use of patient-important outcomes in critical care trials.

**Electronic supplementary material:**

The online version of this article (doi:10.1186/s13613-017-0243-z) contains supplementary material, which is available to authorized users.

## Background

The paternalistic model of patient care has also encompassed the field of research in critical care for many years. To change this paradigm, some clinicians and researchers recently advocated for “the patient at the center” of medical decision making. They suggested recommending interventions, not when the magnitude of the effect was “clinically relevant” but when it was “patient important” [1]. The notion of “Patient-important” sheds light on the individual clinical encounter and the preeminence of patient’s value and preferences within that encounter. In clinical research, a patient-important outcome has been previously defined as: “a characteristic or variable that reflect how a patient feels, functions or survives” [2, 3].

The principal goal of implementing intensive care units (ICU) was to save the life of critically ill patients (i.e., decrease mortality). This goal has been reached in many clinical situations such as septic shock or acute respiratory failure, owing to progress in symptomatic and etiologic treatments of shock and in mechanical ventilation [4, 5]. Although this goal remains a major objective for intensivists, other priorities have emerged. In particular, the importance of assessing mean and long-term outcome in survivors has been underlined [6]. Critical illness is indeed associated with a wide array of long-term sequelae (physical and psychical) that impact functional status and quality of life [7–11]. To account for the patient perspective, clinical decision making by ICU physicians now pursues the goal of improving mean and long-term outcomes in survivors in addition to increasing their chance of survival. In case these goals cannot be reached, an alternative goal is improving the quality of death and dying in ICU.

In addition to mortality, assessing mean to long-term outcomes (after ICU and hospital discharge) could help define the usefulness of an intervention, taking into account what might be relevant and advantageous for the patients [12].

Numerous questions around outcomes used in randomized controlled trials (RCTs) in critically ill patients led us to conduct this systematic review. The core question of this review was guided, however, by patients’ priorities. We chose to define patient-important outcomes according to these patients’ priorities (survival, quality of life, functional, cognitive and neurological performance assessed after ICU discharge) as it has been done in other fields such as diabetes [13].

The main objective of this systematic review was to investigate whether RCTs in critically ill patients published in 2013 assess the patient-important outcomes.

## Methods

To perform the systematic review on the 1-year period of 2013, we followed the PRISMA (Preferred Reporting Items for Systematic Reviews and Meta-Analysis) statement guidelines [14].

### Outcome classification

Our outcome classification was developed according to previous work on patient-important outcome in various medical domains [3, 13, 15].

A scientific committee [including three intensivists (SG, J-DR and DD) and 1 methodologist (FT) particularly involved in designing and conducting RCTs in critically ill patients] established a classification of outcome categories relevant to ICU trials.

The experts identified six outcome categories:

*Patient-important outcomes* that included two entities: on the one hand, mortality at any time and on the other, quality of life, functional/cognitive/neurological outcomes assessed after ICU discharge.

*Clinical outcomes in ICU and hospital* organ failure, complication/adverse outcomes (for instance: drug induced skin reaction or hypotension during renal replacement therapy), healthcare-associated outcomes (nosocomial pneumonia, catheter-related infections), delirium, clinical events (such as venous thromboembolism, myocardial infarction), pain (in ICU), anxiety (in ICU), conscience level, return to spontaneous circulation, muscle strength/circumference, sleep duration, National Institute of Health Stroke Score (NIHSS) (for acute phase of stroke), clinical response to antibiotics, dyspnea (in ICU), noninvasive ventilation tolerance.

*Biological/physiological/radiological outcomes* such as brain natriuretic peptide (BNP), neutrophil gelatinase-associated lipocalin (NGAL), total lung capacity, chest X-ray severity score.

*Care provider decision-related outcomes* e.g., mechanical ventilation duration, length of stay, antibiotic exposure, volume of fluid resuscitation, intubation or reintubation, number of gastric tubes for aspiration, sedation exposure (dose/time), renal replacement therapy, ICU readmission, noninvasive ventilation, tracheostomy, transfusion, use of a prokinetic agent, need for surgery, dose of local anesthesia, hospital discharge disposition.

*Care performance outcomes* care procedure quality and noise/light exposure.

*Other outcomes* family satisfaction, physician/nurse or other provider satisfaction, cost/charges, withholding/withdrawal of care, patient judgment about his readiness to discharge, workload for staff team, compliance to a care protocol, medicolegal conflict.

Besides, for primary outcomes, we defined a “surrogate outcome” as an outcome measuring a substitute for some other variable (e.g., a biomarker intended to substitute for a clinical endpoint) [2].

### Article eligibility criteria

Articles were eligible if they met the following criteria: article published between January 2013 and December 2013; reporting an RCT involving critically ill adult patients (i.e., adults hospitalized in ICU); written in English.

We considered only the first report of the trial results and trial extension follow-up, i.e., we excluded articles reporting post hoc analyses and sub-analyses of RCTs. Indeed, our aim was to focus on the RCTs’ initial objective. We also excluded phase II studies, pilot studies and physiological crossover studies because studies at this stage of clinical research are expected to explore mainly physiological and feasibility outcomes.

### Search strategy and article selection

Main literature search (for the January 2013 to December 2013 period) was conducted on the July 16, 2014, in MEDLINE (via Pubmed^®^) to identify eligible articles indexed between January 2013 and December 2013. The search strategy relied on two algorithms, one dedicated to articles indexed with Mesh terms and the other dedicated to articles not indexed (at the time of the search), using exclusively free text. Terms related to intensive care were combined with terms related to RCTs. Details regarding the literature search strategy and the terms used are provided in Additional file 1.

Two senior intensivists (SG and JM) independently screened the titles and abstracts for the eligibility criteria, to identify articles to be read in full text. Definite article selection was only achieved after examination of the full text confirmed that inclusion criteria were met.

### Data collection

A standardized extraction form (available from the corresponding author) was established from a literature review and a priori discussion. This extraction form was pretested by two authors (SG and JM) independently, in a set of ten articles. This test enabled to identify items needing rewording to avoid any confusion. Disagreements were discussed with an epidemiologist (last author, FT), to ensure similar understanding. Once all litigious points were settled, two reviewers (SG and JM) independently extracted the following data from the selected articles (using the full text and the Additional file 1): general data (funding source, geographical origin, topic, number of centers), methods (intervention assessed, study design, randomization design), quality assessment (by use of the risk of bias tool [16]), trial characteristics (inclusion period, length of follow-up, number of randomized patients) and outcomes (time from randomization to assessment for primary outcome, type and characteristic of all outcomes, see paragraph above).

For all articles included in the systematic review, disagreements between the two reviewers (SG and JM) were resolved by consensus. In case of persistent disagreement, arbitration by a third reviewer (FT) settled the discrepancy.

### Statistical analyses

A 1-year time frame was chosen because it yielded a convenient study sample. Because of the significant lag in study indexation, the closest complete year available at the time of the literature search was 2013.

Continuous variables are described with median and interquartile range (IQR). Categorical variables are described with frequencies and percentages. Distribution of outcomes into the six categories (patient-important outcomes, clinical outcomes, bio/physio/radio outcomes, care provider decision-related outcomes, care performance outcomes and others outcomes) is presented as radar plot. For primary and secondary outcomes, distribution is presented for all outcomes and according to three major topics.

Trial characteristics associated with the presence of at least one patient-important outcome (primary or secondary outcome) were identified in univariate analysis, using Chi-square test or Fisher’s test for categorical variables and Student’s test or Wilcoxon’s test for continuous variables.

Inter-reviewer agreement was measured by the kappa statistic for the following categorical variables: funding source, geographical origin, intervention assessed, unit of randomization and primary outcome category.

Statistical analysis was performed with GraphPad Prism 5 (GraphPad Software, San Diego, USA) and SAS version 9.3 (SAS Institute Inc, Cary, NC).

## Results

### Selection of articles and inter-reviewer agreement

**Fig. 1:**
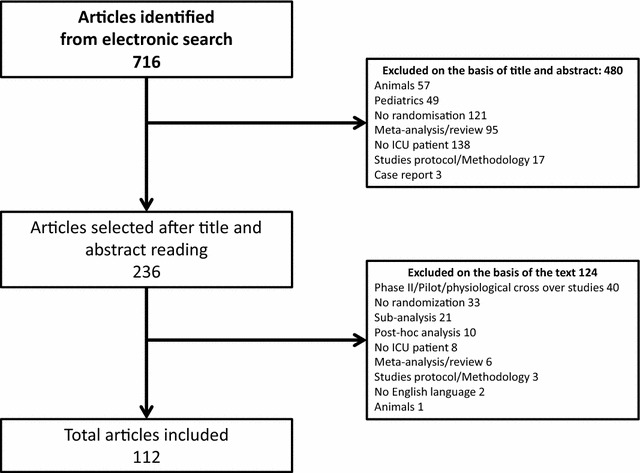
PRISMA flow diagram

The electronic search identified 716 articles. Four hundred and eighty were excluded on the basis of the title and abstract, and 124 after reading the full text. A total of 112 articles reporting RCTs in critically ill patients were finally included and analyzed (see Fig. 1 for PRISMA flow diagram).

Inter-reviewer agreement before consensus for categorical variables was very good [17]: The median kappa value was 0.89 [0.83–0.95] for funding source, 0.99 [0.96–1.00] for geographical origin, 0.83 [0.68–0.99] for unit of randomization and 0.95 [0.91–0.99] for primary outcome category; and good for intervention assessed (median kappa 0.74 [0.65–0.82]).

### Characteristics of the 112 RCTs

Table 1 summarizes RCTs characteristics. Mechanical ventilation (27%), sepsis (18%) and nutrition (17%) were the most common topics of these trials. Therapeutic strategy (41%), drug (33%) and device (11%) evaluation were the most frequent types of intervention.

**Table 1:** Characteristics of RCTs in critically ill adult patients

Characteristic	RCTs without any patient-important outcomes no. (%)	RCTs reporting at least one patient-important outcome no. (%)	*P* value
Total–*n*	39	73	
*Funding*			0.04
Public	14 (36)	42 (57)	
Industry	6 (15)	8 (11)	
Both public and private	18 (46)	10 (14)	
Not reported or unclear	1 (3)	13 (18)	
*Geographical area*			
Europe	12 (31)	34 (47)	
Asia	18 (46)	19 (26)	
North America	4 (10)	16 (22)	
Oceania	3 (8)	9 (12)	
South America	2 (5)	8 (11)	
Africa	0 (0)	5 (7)	
International (>1 country)	0 (0)	18 (25)	
*Number of center(s)*			0.005
Monocenter	29 (74)	33 (45)	
Multicenter	8 (21)	38 (52)	
Unclear	2 (5)	2 (3)	
*Sample size*			0.01
<50	16 (41)	16 (22)	
50–100	7 (18)	14 (19)	
100–150	6 (15)	5 (7)	
150–500	7 (18)	20 (27)	
>500	3 (8)	18 (25)	
*Topic of the study* ^a^			
Mechanical ventilation	14 (36)	16 (22)	0.011
Sepsis	2 (5)	19 (26)	0.007
Nutrition	5 (13)	14 (19)	0.39
Infection	8 (21)	10 (14)	
Hemodynamics	2 (5)	4 (5)	
ARDS	2 (5)	7 (10)	
Cardiac arrest	2 (5)	4 (5)	
Trauma	3 (8)	3 (4)	
Sedation	2 (5)	1 (1)	
Acute kidney injury	1 (3)	3 (4)	
Pain	2 (5)	2 (3)	
Neurocritical care	1 (3)		
Hematological issue	1 (3)	2 (3)	
Rehabilitation/physical and/or cognitive therapy	2 (5)		
Metabolic disorder	1 (3)		
Burns	0 (0)	1 (1)	
ECMO	0 (0)	1 (1)	
Electric muscle stimulation	1 (3)		
Music	1 (3)		
*Type of intervention*			0.37
Therapeutic strategy	14 (36)	32 (44)	
Drug	13 (33)	24 (33)	
Device	6 (15)	7 (10)	
Monitoring	2 (5)	1 (1)	
Diagnostic strategy	0 (0)	4 (5)	
Other	4 (10)	5 (7)	
*Unit of randomization*			0.74
Patient	35 (90)	67 (92)	
Service	2 (5)	3 (4)	
Time	1 (3)	1 (1)	
Other	1 (3)	2 (3)	
*Type of trial*			1.00
Superiority	38 (97)	70 (96)	
Equivalence or non-inferiority	1 (3)	3 (4)	
*Follow-up*			
Fixed time point	22 (56)	41 (56)	
Median months [IQR]	0.5 [0.5–1]	3 [1–9]	0.0002
ICU	7 (18)	7 (10)	
Hospital	3 (8)	12 (16)	
Unreported	7 (18)	13 (18)	

The numbers in parentheses mean the percentage; ECMO: extracorporeal membrane oxygenation

A study can appear in more than one row for geographical area

^a^One study could have more than one topic

Follow-up period was defined until a fixed time point for 44 (39%) RCTs (median [IQR] 3 [1–6] months of follow-up), until ICU discharge for 14 (12%) RCTs and until hospital discharge for 15 (13%) RCTs. Follow-up period was unclear for 39 (35%) RCTs.

### Quality assessment

**Fig. 2:**
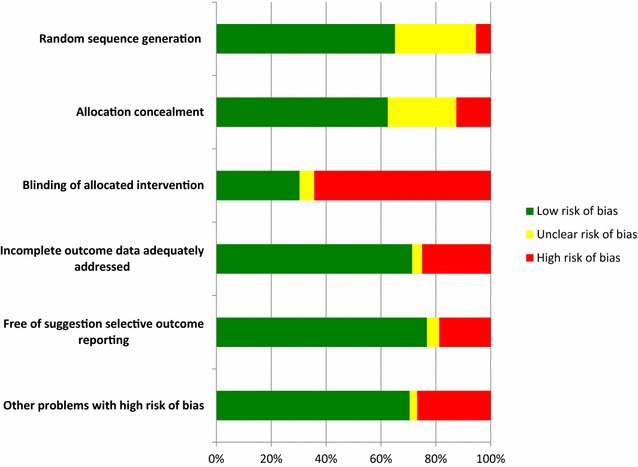
RCTs quality assessment by risk of bias tool [16]. Methodological quality of the trials included in the systematic review assessed by six points: random sequence generation, allocation concealment, blinding of allocation intervention, incomplete data adequately addressed, free of suggestion selective outcome reporting and other problems. Horizontal axis represents the ratio (%) distribution among “low risk of bias” (*green*), “high risk of bias” (*red*) and “unclear risk of bias” (*yellow*)

Quality of the trials using the risk of bias tool is shown in Fig. 2. The absence of blinding of allocated intervention was the most frequent methodological component introducing a high risk of bias.

### Primary outcomes

**Fig. 3:**
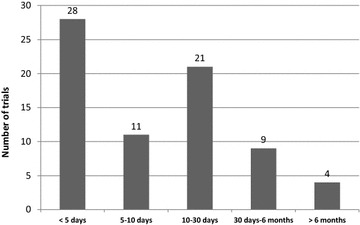
Time from randomization to assessment of primary outcome. This figure represents the distribution of the time from randomization to assessment of primary outcome for the 73 RCTs that assessed the primary outcome after a fixed time point

Seventy-three (65%) RCTs assessed the primary outcome after a median [IQR] fixed time point of 7 [2–28] days (from randomization) and only 13 (12%) assessed the primary outcome beyond 30 days (Fig. 3). The other RCTs assessed the primary outcome at ICU discharge (*n* = 25, 22%) or hospital discharge (*n* = 9, 8%). Five (4%) did not specify the time from randomization to primary outcome assessment.

Among the 112 primary outcomes, 27 (24%) were patient-important outcomes. Most of them were mortality (21/27, 78%) and only 6/27 (22%) were quality of life, functional/cognitive/neurological outcomes assessed after ICU discharge (functional disability = 4; quality of life = 2).

**Fig. 4:**
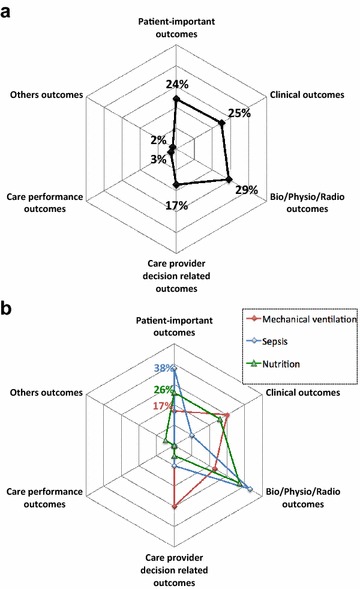
Distribution of primary outcomes. **a** Distribution of 112 primary outcomes, percentage of primary outcomes by outcome category, **b** distribution of primary outcomes according to three major topics (mechanical ventilation, sepsis and nutrition), percentage of primary outcomes by outcome category

Among the 21 mortality outcomes, 18 were assessed after a fixed time point of 28 [28–60] days. The other three were assessed at ICU discharge. Among the six quality of life, functional/cognitive/neurological outcomes, two were assessed at hospital discharge and four after a fixed time point (6, 12, 12, 14 months). Figure 4 shows the distribution of the 112 primary outcomes and according to the three major topics (mechanical ventilation, sepsis, nutrition). Besides, 45 (40%) primary outcomes were surrogate endpoints.

### Secondary outcomes

Among 598 secondary outcomes identified, 133 (22%) were patient-important outcomes. Most of them were mortality (92/133, 69%) and only 41 (31%) were quality of life, functional/cognitive/neurological outcomes assessed after ICU discharge (quality of life = 20, functional disability = 14; neurological/cognitive performance = 5; handicap = 1; post-traumatic stress = 1).

Among the 92 mortality outcomes, 43 were assessed after a fixed time point of 28 [28–90] days. The others were assessed at ICU discharge (*n* = 26) or at hospital discharge (*n* = 23). Among the 41 quality of life, functional/cognitive/neurological outcomes, 37 were assessed at a fixed time point of 365 [319–380] days and four at hospital discharge.

**Fig. 5:**
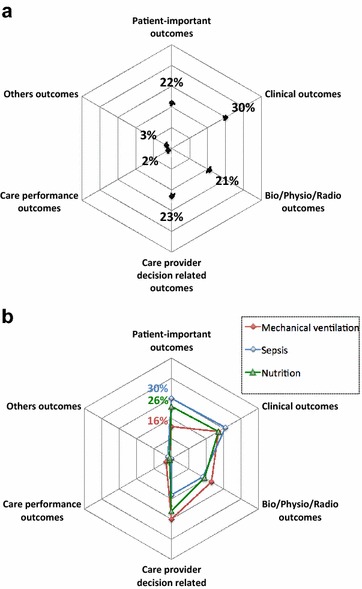
Distribution of secondary outcomes. **a** Distribution of 598 secondary outcomes, percentage of secondary outcomes by outcome category, **b** distribution of secondary outcomes according to three major topics (mechanical ventilation, sepsis and nutrition), percentage of secondary outcomes by outcome category

Figure 5 shows the distribution of the 598 secondary outcomes and according to the three major topics (mechanical ventilation, sepsis, nutrition).

### Trial characteristics associated with the presence of at least one patient-important outcome (primary or secondary)

Among the 112 RCTs, 73 (65%) reported at least one patient-important outcome (primary or secondary outcomes) but only 11 (10%) reported at least one quality of life, functional/cognitive/neurological outcomes assessed after ICU discharge. Characteristics of these RCTs are provided in Table 1.

## Discussion

We found that, during the 1-year survey period of RCTs performed in critically ill patients, a minority of outcomes used in these RCTs were patient-important outcomes. They accounted for 24 and 22% of primary and secondary outcomes, respectively. Mortality accounted for the vast majority of reported patient-important outcomes, whereas other patient-important outcomes (such as quality of life, functional/cognitive/neurological outcomes assessed after ICU discharge) were scarcely used (7% of all outcomes). Moreover, only 10% of surveyed RCTs reported at least one patient-important outcome besides mortality (quality of life, functional/cognitive/neurological outcomes assessed after ICU discharge). This is at striking contrast with clinical decision making of ICU physicians, which is felt to be in line with these crucial outcomes. In addition, we were cautious to retain only those RCTs for which patient-important outcomes were reasonably expected. Indeed, studies for which patient-important outcomes were less likely to be present (phase II studies, pilot studies and physiological crossover studies) were not included.

Our study is the first to explore the place of patient-important outcomes and how well they are reported in RCTs in critically ill patients. We derived the definition of patient-important outcome for critically ill patients from the definition used in other fields [3, 13, 15]. Mortality is obviously essential, and we considered that quality of life, functional/cognitive/neurological outcomes assessed after ICU discharge also qualified for patient-important outcome. Indeed, the potential adverse consequences of an ICU stay are best evaluated after ICU discharge and even more after hospital discharge since critical illness is associated with long-term sequelae. Survivors of critical care experience profound changes in their lives because of many forms of deficit in one or more domains [18] of physical [19, 20], psychological [11, 21, 22] or cognitive functioning [22–25]. These numerous symptoms led to define the new entity of “post-intensive care syndrome” [26].

This study is the first to use a definition of patient-important outcomes for critically ill patients. This definition is open to criticism on two points. Firstly, it includes exogenous measures of symptoms that may not perfectly capture how patients feel or how symptoms impact their overall quality of life. We could have restricted the definition to patient-reported outcomes [27]. Nevertheless, doing this, the message of this study would have been the same. Indeed, among the 27 primary outcomes, which were patient-important outcomes, four were classified as functional disability and two of those were not patient-reported outcomes. Secondly, besides mortality outcomes, we chose to restrict the patient-important outcomes to the post-ICU period (leaving out pain, anxiety and dyspnea which might have occurred during the ICU stay). However, in the present systematic review, “pain, anxiety and dyspnea in ICU” accounted for only two (1.8%) primary outcomes and four (0.7%) secondary outcomes. If we had considered these outcomes as patient-important outcomes, the results of this study would have been very similar.

To perform this systematic review, we developed an outcome classification relevant to the context of critical care, involving six categories (patient-important outcomes, clinical outcomes in ICU and hospital, biological/physiological/radiological outcomes, care provider decision-related outcomes, care performance outcomes and others). A systematic review is the first step to establish a core outcome set and our outcome classification could help researchers to clarify the place of patient-important outcomes in core outcome sets for future RCTs in critically ill patients. To date, there is no taxonomy of outcomes studied in critically ill patients, nor core outcome set. This may cause inconsistencies in outcome reports and difficulties in comparing these outcomes across studies and to combine them in systematic reviews and meta-analyses [28]. With the aim to facilitate the development and application of agreed standardized sets of outcomes, the Core Outcome Measures in Effectiveness Trials (COMET) initiative was initiated in [29]. In the field of critical care, the Core Outcomes in Ventilation Trials (COVenT) is in progress [30]. This systematic review can be the first step to develop other core outcome sets in other topics of critical care and to establish a core outcome set involving patients’ opinion for future RCTs.

An inherent limitation of a systematic review of published trials is that it is performed at a given period (here 2013). The search led to identify 112 eligible RCTs, which provides a very large panel of ICU trials and thus robust information on the prevalence of patient-important outcomes in RCTs in critically ill patients. Many systematic reviews rely issues on a 1-year literature search [31, 32]. Our goal was to capture the most recent practices in trials as the literature on patient-important outcomes in other medical field and the growing interest for the patients’ perspective may have had an impact.

Patient-important outcomes are gaining wide acceptance in some fields of clinical research [33–35]. Additionally, a recent survey from 2036 patients with diabetes showed that most of them (>75%) chose patient-important outcomes rather than HbA1c as their first choice for a trial primary outcome [36]. Patients understand the reality of their condition and disease’s impact on their lives better than physicians can do [37]. James Lind Alliance in the UK [38, 39] and the Patient-Centered Outcome Research Institute in the USA [40–43] showed the mismatch between questions patients and clinicians needed an answer for on the one hand and those that were investigated by researchers on the other. This led some opinion leaders to call for a patient revolution [44]. Patients who survive after a critical illness may experience many sequelae after ICU or hospital discharge. In our study, only 10% of RCTs reported at least one non-mortality patient-centered outcome assessed after ICU discharge. It seems therefore desirable that more long-term outcomes be assessed in ICU studies.

Reasons explaining this small percentage of RCTs assessing patient-important outcomes and in particular the impressive scarcity of outcomes assessed after ICU discharge are diverse. One of them is the difficulty to ascertain mean and long-term follow-up of patients. The preference of researchers and funding agencies for rapidly obtained results favors short-term outcomes. This could be a shortcoming since paradoxical short- and long-term effects after certain interventions have been described [45, 46]. For instance, after acute myocardial infarction flecainide decreased arrhythmias but has been associated with increased mortality [45]. In critical illness, growth hormone improved nitrogen balance but has been also associated with increased mortality [47]. Moreover, many proposed short-term endpoints in critical care have not been formally evaluated for surrogacy [48] precluding any strong conclusion on the effect on patient-important outcomes. For example, acute organ dysfunction in ICU does not appear to have significant long-term implications for patient-important outcomes [49]. In our systematic review, we found that 40% of primary outcomes were surrogate endpoints.

To promote the assessment of patient-important outcomes, patients’ follow-up should be extended but this can be hampered by logistical issues (organization of phone call, medical consultation, etc.) which can considerably increase the costs of the study. As a result, we found that patients’ follow-up was short since only 12% of trials assessed primary outcomes beyond 30 days from randomization. Patient-important outcomes were initially promoted to evaluate outcomes of chronic diseases [13, 15] for which patients’ follow-up is easier to perform because the patient is cared for by the same medical team. This situation is quite different for critically ill patients who are often cared for by a different team after ICU discharge. The advent of post-ICU consultation [50] could foster a better assessment of patient-important outcomes by intensivists and researchers in the field.

Additionally, lengthy technical questionnaires are usually used to assess patient-reported outcomes (i.e., quality of life or functional status) after ICU discharge. This often leads to a high proportion of non-responders that renders interpretation more difficult [51]. The question of the applicability to the ICU setting of the tools used is also raised by ICU experts: “Are existing instruments suitable for capturing important nuances of post-ICU sequelae or should disease-specific instruments be captured” [6].

The decisions intensivists make at the bedside aim at both saving lives and preserving—at best—their patients’ prior quality of life. Medical research and especially RCTs should help them to better evaluate the efficacy of their interventions (drug administration, therapeutic strategy implementation or device use) on these relevant issues.

 Our results indicate that outcomes of many RCTs remained too often centered on physiological criteria (oxygenation or hemodynamic stabilization for instance) or assessed mortality as sole outcome of importance for patients. This has two pitfalls: quality of life of survivors is not assessed, and given the noticeable improvement of vital prognosis in a number of ICU situations (ARDS for example), many interventional studies using mortality as primary outcome have been negative in recent years, mandating the use of alternative outcome measures, such as patient-important ones [48, 52, 53].

## Conclusion

 Our study shows that only a small number of primary outcomes measures in recent RCTs performed in the ICU are patient-important outcomes. To better address patient needs, researchers should take the crucial post-ICU period into account in the design of future RCTs. This is one of the challenges for future ICU research. This paradigm shift would be in the interest of patients.

## Additional file

**Additional file 1.** Terms used for the literature search strategy.

## Abbreviations

ICU: intensive care units
RCT: randomized controlled trial
PRISMA: Preferred Reporting Items for Systematic Reviews and Meta-Analysis
ARDS: acute respiratory distress syndrome
NIHSS: National Institute of Health Stroke Score
BNP: brain natriuretic peptide
NGAL: neutrophil gelatinase-associated lipocalin
