# Supplementary material for: Patient-important outcomes in randomized controlled trials in critically ill patients: a systematic review
Source: Ann Intensive Care. 2017 Mar 7;7:28. doi: 10.1186/s13613-017-0243-z (PMC5340787; doi:10.1186/s13613-017-0243-z)
Supplement: Supplementary file 1 — Additional file 1. Terms used for the literature search strategy. [file 13613_2017_243_MOESM1_ESM.docx]

**Supplementary appendix**

LITERATURE SEARCH STRATEGY 2

INFORMATION ABOUT THE 112 INDIVIDUAL STUDIES 4

# LITERATURE SEARCH STRATEGY

- - **Search strategy n°1 dedicated to articles indexed with MeSH terms**

((Intensive Care Units[MeSH Terms] **OR** critical care[MeSH Terms] **OR** critical illness[MeSH Terms]) **OR** (High flow nasal oxygen[Title] **OR** Sepsis[Title] **OR** Septic shock[Title] **OR** Extubation[Title] **OR** Cardiac arrest[Title] **OR** ECMO[Title] **OR** Extracorporeal membrane oxygenation[Title] **OR** Extra corporeal membrane oxygenation[Title] **OR** Ventilator-associated[Title] **OR** Ventilated[Title] **OR** Mechanical ventilation[Title] **OR** Lung injury[Title] **OR** ARDS[Title] **OR** Acute respiratory distress syndrome[Title] **OR** resuscitation[Title] **OR** critical illness[Title] **OR** critically ill[Title] **OR** critical care[Title] **OR** intensive care[Title] **OR** icu[Title]))

**AND**

(("randomized controlled trial"[Publication Type] **OR** "double blind method"[MeSH Terms]) **OR** (randomisation[Title/Abstract] **OR** randomised[Title/Abstract] **OR** randomization[Title/Abstract] **OR** randomized[Title/Abstract] **OR** random[Title/Abstract] **OR** randomly[Title/Abstract] **OR** allocated[Title/Abstract] **OR** allocation[Title/Abstract]))

**AND** humans[MeSH Terms]

**AND** adult[MeSH Terms]

**AND** Publication date from 2013/01/01 to 2013/12/31

**AND** English

- - **Search strategy n°2 dedicated to articles not yet indexed with MeSH terms**

(High flow nasal oxygen[Title] **OR** Sepsis[Title] **OR** Septic shock[Title] **OR** Extubation[Title] **OR** Cardiac arrest[Title] **OR** ECMO[Title] **OR** Extracorporeal membrane oxygenation[Title] **OR** Extra corporeal membrane oxygenation[Title] **OR** Ventilator-associated[Title] **OR** Ventilated[Title] **OR** Mechanical ventilation[Title] **OR** Lung injury[Title] **OR** ARDS[Title] **OR** Acute respiratory distress syndrome[Title] **OR** resuscitation[Title] **OR** critical illness[Title] **OR** critically ill[Title] **OR** critical care[Title] **OR** intensive care[Title] **OR** icu[Title]

**AND**

(randomisation[Title/Abstract] **OR** randomised[Title/Abstract] OR randomization[Title/Abstract] **OR** randomized[Title/Abstract] OR random[Title/Abstract] **OR** randomly[Title/Abstract] **OR** allocated[Title/Abstract] **OR** allocation[Title/Abstract])

NOT humans[MeSH Terms]

NOT animals[MeSH Terms]

**AND** Publication date from 2013/01/01 to 2013/12/31

**AND** English

NOT humans and NOT animals have been added in the second search strategy to exclude references indexed by Mesh terms for which either humans or animals should be necessarily present.

#

# INFORMATION ABOUT THE 112 INDIVIDUAL STUDIES

| Author | Journal | Type of intervention | Patients  n | Follow-up | Primary outcome |
| --- | --- | --- | --- | --- | --- |
| Mahmoodpoor A, et al. | Acta Med Iran | DEVICE | 96 | Unclear | Healthcare associated infections |
| Chen Y-Y, et al. | Am J Crit Care | OTHER | 278 | ICU discharge | Invasive equipment exposure |
| Annane D, et al. | Am J Respir Crit Care | DIAGNOSTIC | 411 | 6 months | 29-180 day mortality |
| Kruger P, et al. | Am J Respir Crit Care | DIAGNOSTIC | 250 | 3 months | Biological or pharmacological |
| Needham DM, et al. | Am J Respir Crit Care | CURATIVE | 349 | 12 months | Functional disability |
| Chaari A, et al. | Am J Ther | CURATIVE | 44 | ICU discharge | Healthcare associated infections |
| Goepfert MS, et al. | Anesthesiology | MONITORING | 100 | Unclear | ICU length of stay |
| Kaukonen K-M, et al. | BMC Infect Dis | DIAGNOSTIC | 59 | Unclear | Biological or pharmacological |
| Needham DM, et al. | BMJ | CURATIVE | 951 | 12 months | Quality of life |
| Annane D, et al. | BMJ | PREVENTIVE | 62 | Hospital discharge | Antibiotic exposure |
| Guoshou Z, et al. | Cell Biochem Biophys | DIAGNOSTIC | 50 | ICU discharge | Radiological score |
| Chen J, et al. | Chin Med J | CURATIVE | 58 | Unclear | 28-day mortality |
| Liu L, et al. | Chin Med J | DEVICE | 48 | Unclear | Duration of mechanical ventilation |
| Yang Y-L, et al. | Chin Med J | CURATIVE | 65 | 1 month | 28-day mortality |
| Dumas F, et al. | Circulation | CURATIVE | 2496 | 12 months | 181+ mortality |
| Freese JP, et al. | Circulation | DEVICE | 987 | Hospital discharge | In-hospital mortality |
| Dulhunty JM, et al. | Clin Infect Dis | CURATIVE | 60 | 1 month | Biological or pharmacological |
| Durante-Mangoni E, et al. | Clin Infect Dis | DIAGNOSTIC | 210 | 3 months | 29-180 day mortality |
| Barros KV, et al. | Clin Nutr | DIAGNOSTIC | 53 | ICU discharge | Biological or pharmacological |
| Majid HA, et al. | Clin Nutr | DIAGNOSTIC | 47 | Unclear | Biological or pharmacological |
| Bakiner O, et al. | Crit Care | CURATIVE | 20 | Unclear | Biological or pharmacological |
| Bechir M, et al. | Crit Care | DIAGNOSTIC | 48 | 3 months | Volume of fluid resuscitation |
| Brunner R, et al. | Crit Care | DIAGNOSTIC | 38 | 1 month | Polyneuromyopathy |
| Denehy L, et al. | Crit Care | CURATIVE | 150 | 12 months | Functional disability |
| Gando S, et al. | Crit Care | DIAGNOSTIC | 60 | 1 month | Biological or pharmacological |
| Ornico SR, et al. | Crit Care | CURATIVE | 40 | Hospital discharge | Intubation or re-intubation |
| Robinson S, et al. | Crit Care | DIAGNOSTIC | 78 | ICU discharge | Biological or pharmacological |
| Wittbrodt P, et al. | Crit Care | DIAGNOSTIC | 705 | 12 months | Quality of life |
| Wu J, et al. | Crit Care | DIAGNOSTIC | 367 | 1 month | 28-day mortality |
| Cordoba-Izquierdo A, et al. | Crit Care Med | DEVICE | 25 | Unclear | Sleep electrophysiology |
| Heyland DK, et al. | Crit Care Med | CURATIVE | 1059 | Unclear | Feasibility/Compliance to protocol |
| Oliveira CF, et al. | Crit Care Med | OTHER | 97 | Hospital discharge | Antibiotic exposure |
| Ricard J-D, et al. | Crit Care Med | CURATIVE | 266 | 1 month | Complications/adverse outcome |
| Wang J, et al. | Crit Care Nurse | CURATIVE | 121 | Unclear | Duration of mechanical ventilation |
| Lee MY, et al. | Diabetes Care | DIAGNOSTIC | 20 | Unclear | Biological or pharmacological |
| Van Herpe T, et al. | Diabetes Care | OTHER | 300 | Unclear | Biological or pharmacological |
| Deliberato RO, et al. | Diagn Microbiol Infect Dis | PREVENTIVE | 81 | Unclear | Antibiotic exposure |
| Aydemir H, et al. | Epidemiol Infect | DIAGNOSTIC | 43 | Hospital discharge | Clinical response to antibiotic treatment |
| Maa S-H, et al. | Evid Based Complement Alternat Med | OTHER | 110 | Unclear | Physiological data |
| Liu X-L, et al. | Exp Ther Med | DIAGNOSTIC | 37 | 1 month | Biological or pharmacological |
| Jones CU, et al. | Heart Lung | DEVICE | 15 | Unclear | Physiological data |
| Rugeles S-J, et al. | Indian J Crit Care med | CURATIVE | 115 | ICU discharge | Incidence of acute organ failure |
| Salgado CD, et al. | Infect Control Hosp Epidemiol | DEVICE | 650 | Hospital discharge | Healthcare associated infections |
| Berry AM, et al. | Intensive Crit Care Nurs | DIAGNOSTIC | 398 | Unclear | Biological or pharmacological |
| Airapetian N, et al. | Intensive Care Med | CURATIVE | 118 | Unclear | Success venous canulation |
| Bein T, et al. | Intensive Care Med | CURATIVE | 79 | 2 months | Ventilator-free days |
| Bouza E, et al. | Intensive Care Med | CURATIVE | 78 | Hospital discharge | Healthcare associated infections |
| Hernandez G, et al. | Intensive Care Med | CURATIVE | 195 | Hospital discharge | Duration of mechanical ventilation |
| Joannes-Boyau O, et al. | Intensive Care Med | CURATIVE | 140 | 3 months | 28-day mortality |
| Lellouche F, et al. | Intensive Care Med | DEVICE | 249 | ICU discharge | Intubation or re-intubation |
| Vignon P, et al. | Intensive Care Med | DEVICE | 407 | 3 months | Venous thromboembolism |
| Ahmed SM, et al. | Int J Crit Illn Inj Sci | DIAGNOSTIC | 300 | Unclear | Biological or pharmacological |
| Ponce D, et al. | Int Urol Nephrol | CURATIVE | 143 | 2 months | In-hospital mortality |
| Santamaria N, et al. | Int Wound J | DEVICE | 440 | Unclear | Acquired pressure ulcer |
| Serra R, et al. | Int Wound J | DIAGNOSTIC | 21 | Unclear | Acquired pressure ulcer |
| Abdar ME, et al. | Iran J Nurs Midwifery Res | CURATIVE | 132 | Unclear | Level of consciousness |
| Yazdannik AR, et al. | Iran J Nurs Midwifery Res | CURATIVE | 50 | Unclear | Physiological data |
| Kouchek M, et al. | Iran J Pharm Res | CURATIVE | 40 | Unclear | Pain |
| Annane D, et al. | JAMA | DIAGNOSTIC | 2857 | 3 months | 28-day mortality |
| Chlan LL, et al. | JAMA | OTHER | 373 | 1 month | Anxiety / stress / depression |
| Doig GS, et al. | JAMA | CURATIVE | 1372 | 2 months | 29-180 day mortality |
| Harris AD, et al. | JAMA | DEVICE | 26180 | Unclear | Healthcare associated infections |
| Jubran A, et al. | JAMA | CURATIVE | 316 | 12 months | Duration of mechanical ventilation |
| Mentzelopoulos SD, et al. | JAMA | DIAGNOSTIC | 300 | 2 months | 29-180 day mortality |
| Morelli A, et al. | JAMA | DIAGNOSTIC | 154 | 1 month | Physiological data |
| Opal SM, et al. | JAMA | DIAGNOSTIC | 1985 | 12 months | 28-day mortality |
| Papazian L, et al. | JAMA | DIAGNOSTIC | 300 | 3 months | 28-day mortality |
| Reignier J, et al. | JAMA | MONITORING | 452 | 3 months | Healthcare associated infections |
| Young D, et al. | JAMA | CURATIVE | 909 | 24 months | 28-day mortality |
| Su C-P, et al. | J adv nurs | OTHER | 28 | Unclear | Sleep electrophysiology |
| Narahara H, et al. | J Anesth | DIAGNOSTIC | 25 | 1 month | Biological or pharmacological |
| Sanaie S, et al. | J Cardiovasc Thorac Res | DIAGNOSTIC | 40 | Unclear | Biological or pharmacological |
| Niven DJ, et al. | J Crit Care | CURATIVE | 216 | ICU discharge | Duration of mechanical ventilation |
| Vahdat Shariatpanahi Z, et al. | J Crit Care | OTHER | 32 | Unclear | Physiological data |
| Saxena A, et al. | J Nat Sci biol Med | DIAGNOSTIC | 36 | Unclear | Complications/adverse outcome |
| Condessa RL, et al. | J Physiother | CURATIVE | 92 | ICU discharge | Duration of mechanical ventilation |
| Aghadavoudi O, et al. | J Res Med Sci | DIAGNOSTIC | 60 | ICU discharge | Complications/adverse outcome |
| Marasco SF, et al. | J Am Coll Surg | CURATIVE | 46 | 6 months | Duration of mechanical ventilation |
| Jaruratanasirikul S, et al. | J Med Assoc Thai | DIAGNOSTIC | 9 | Unclear | Biological or pharmacological |
| Jaruratanasirikul S, et al. | J Med Assoc Thai | CURATIVE | 9 | Unclear | Biological or pharmacological |
| Zhang H, et al. | J Tradit Chin Med | DIAGNOSTIC | 64 | Unclear | Incidence of acute organ failure |
| Puskarich MA, et al. | J Parent Enteral Nutr | CURATIVE | 31 | 12 months | Complications/adverse outcome |
| Williams TA,and al. | J Parent Enteral Nutr | PREVENTIVE | 360 | Hospital discharge | Number of gastric tube aspiration |
| Heidegger CP, et al. | Lancet | CURATIVE | 305 | 1 month | Healthcare associated infections |
| Du Cheyron D, et al. | Nephrol Dial Transplant | MONITORING | 74 | ICU discharge | Incidence of acute organ failure |
| Wei L, et al. | Pak J Med Sci | CURATIVE | 615 | Unclear | Healthcare associated infections |
| Matejec R, et al. | Peptides | DIAGNOSTIC | 16 | Unclear | Biological or pharmacological |
| Falavigna LF, et al. | Physiother Theory Pract | CURATIVE | 25 | Unclear | Functional disability |
| Arima T, et al. | Am J Emerg Med | DEVICE | 121 | Unclear | Time/Difficulty of intubation |
| Schmelzer TM, et al. | Am Surgeon | CURATIVE | 73 | Unclear | Biological or pharmacological |
| Ledgerwood LG, et al. | Ann OtolRhinol Laryngol | DEVICE | 18 | Unclear | Healthcare associated infections |
| Yosef-Brauner O, et al. | Clin Respir J | CURATIVE | 18 | ICU discharge | Functional disability |
| Blackwood B, et al. | J Antimicrob Chemother | DIAGNOSTIC | 445 | Hospital discharge | Healthcare associated infections |
| Giamarellos-Bourboulis EJ, et al. | J Antimicrob Chemother | DIAGNOSTIC | 600 | 1 month | 28-day mortality |
| Hua F, et al. | J Emerg Med | DIAGNOSTIC | 32 | Hospital discharge | Physiological data |
| Huang Y, et al. | J Surg Res | CURATIVE | 30 | ICU discharge | Physiological data |
| Onorati F, et al. | J Thorac Cardiovasc Surg | CURATIVE | 30 | ICU discharge | Physiological data |
| Rogers CA, et al. | J Thorac Cardiovasc Surg | CURATIVE | 184 | 12 months | Hospital length of stay |
| Kirkpatrick AW, et al. | J Trauma Acute Care Surg | CURATIVE | 95 | Hospital discharge | Incidence of acute organ failure |
| Derde LPG, et al. | Lancet Infect Dis | PREVENTIVE | 4861 | Hospital discharge | Healthcare associated infections |
| Page VJ, et al. | Lancet Respir Med | DIAGNOSTIC | 142 | 1 month | Delirium |
| Ferguson ND, et al. | N Eng J Med | CURATIVE | 548 | 2 months | In-hospital mortality |
| Guerin C, et al. | N Eng J Med | CURATIVE | 474 | 3 months | 28-day mortality |
| Heyland D, et al. | N Eng J Med | DIAGNOSTIC | 1223 | 6 months | 28-day mortality |
| Huang SS, et al. | N Eng J Med | CURATIVE | 74256 | Hospital discharge | Healthcare associated infections |
| Jabre P, et al. | N Eng J Med | OTHER | 570 | 3 months | Family satisfaction |
| Kerlin MP, et al. | N Eng J Med | OTHER | 1598 | 3 months | ICU length of stay |
| Nielsen N, et al. | N Eng J Med | CURATIVE | 939 | 6 months | 181+ mortality |
| Young D, et al. | N Eng J Med | CURATIVE | 795 | 1 month | 29-180 day mortality |
| Chancharoenthana W, et al. | Ther Apher Dial | CURATIVE | 28 | Unclear | Biological or pharmacological |
| Huang Z, et al. | Ther Apher Dial | DEVICE | 46 | Unclear | Physiological data |
| Zhao G, et al. | World J Gastroenterol | DIAGNOSTIC | 120 | Hospital discharge | Volume of fluid resuscitation |
